# Microglial re-modeling contributes to recovery from ischemic injury of rat brain: A study using a cytokine mixture containing granulocyte-macrophage colony-stimulating factor and interleukin-3

**DOI:** 10.3389/fnins.2022.941363

**Published:** 2022-07-28

**Authors:** Shirabe Matsumoto, Mohammed E. Choudhury, Haruna Takeda, Arisa Sato, Nanako Kihara, Kanta Mikami, Akihiro Inoue, Hajime Yano, Hideaki Watanabe, Yoshiaki Kumon, Takeharu Kunieda, Junya Tanaka

**Affiliations:** ^1^Department of Neurosurgery, Graduate School of Medicine, Ehime University, Toon, Japan; ^2^Department of Molecular and Cellular Physiology, Graduate School of Medicine, Ehime University, Toon, Japan

**Keywords:** GM-CSF, IL-3, microglia, macrophage, Bcl-xL, brain ischemia

## Abstract

Ischemic stroke is a leading cause of mortality and permanent disability. Chronic stroke lesions increase gradually due to the secondary neuroinflammation that occurs following acute ischemic neuronal degeneration. In this study, the ameliorating effect of a cytokine mixture consisting of granulocyte-macrophage colony-stimulating factor (GM-CSF) and interleukin (IL)-3 was evaluated on ischemic brain injury using a rat stroke model prepared by transient middle cerebral artery occlusion (tMCAO). The mixture reduced infarct volume and ameliorated ischemia-induced motor and cognitive dysfunctions. Sorted microglia cells from the ischemic hemisphere of rats administered the mixture showed reduced mRNA expression of tumor necrosis factor (TNF)-α and IL-1β at 3 days post-reperfusion. On flow cytometric analysis, the expression of CD86, a marker of pro-inflammatory type microglia, was suppressed, and the expression of CD163, a marker of tissue-repairing type microglia, was increased by the cytokine treatment. Immunoblotting and immunohistochemistry data showed that the cytokines increased the expression of the anti-apoptotic protein Bcl-xL in neurons in the ischemic lesion. Thus, the present study demonstrated that cytokine treatment markedly suppressed neurodegeneration during the chronic phase in the rat stroke model. The neuroprotective effects may be mediated by phenotypic changes of microglia that presumably lead to increased expression of Bcl-xL in ischemic lesions, while enhancing neuronal survival.

## Highlights

- A rat brain ischemia model was prepared by transient middle cerebral artery occlusion.
- A cytokine mixture of IL-3 and GM-CSF was administered to the ischemia model.
- The mixture reduced infarct volume and ameliorated motor and cognitive dysfunctions.
- The mixture enhanced expression of the anti-apoptotic factor Bcl-xL in the ischemic lesion.
- The mixture changed microglial phenotypes from pro-inflammatory to tissue-repairing.

## Introduction

Ischemic stroke is the second leading cause of avoidable deaths and the third most common cause of long-term disability worldwide (Johnson et al., 2016). Regarding its pathogenesis, post-ischemic neuroinflammation has been investigated, because inflammatory cascades contribute noticeably to secondary brain damage following the ischemia-induced primary injury (Iadecola and Anrather, 2011). Ischemic events trigger a series of complex cellular responses, which include activation of resident glial cells and recruitment of inflammatory cells from the circulation. Evidence shows that microglia and infiltrated macrophages play critical roles in regulating the immune and inflammatory responses after brain injuries (Perry et al., 2010).

Microglia/macrophages have different phenotypes with divergent functions during the progression of ischemic brain injury (Hu et al., 2012). Microglia/macrophages play divergent roles in ischemic brain regions: one is beneficial, protecting and repairing; and the other is detrimental, pro-inflammatory and destructive. The former cells have often been termed tissue-repairing type cells that are characterized by abundant production of anti-inflammatory and neuroprotective cytokines and growth factors; the latter cells are pro-inflammatory type cells characterized by abundant expression of pro-inflammatory mediators such as interleukin-1β (IL-1β) or reactive oxygen species (ROS) that are potentially neurotoxic (Brifault et al., 2015).

Granulocyte–macrophage colony-stimulating factor (GM-CSF) is a pluripotent cytokine that activates microglia/macrophages and has been shown to have ameliorating effects on brain ischemia (Nakagawa et al., 2006; Schabitz et al., 2008; Li et al., 2013; Dames et al., 2018). Similar to GM-CSF, interleukin-3 (IL-3) stimulates microglia/macrophages (Ganter et al., 1992). Previous reports from our lab showed cumulative ameliorating effects of a cytokine mixture containing GM-CSF and IL-3 against traumatic brain injury (TBI) and Parkinson’s disease (PD). Increased expression of the anti-apoptotic factor Bcl-xL in neuronal cells may be correlated to the beneficial effects of both cytokines (Choudhury et al., 2011; Nishihara et al., 2011).

The current study aimed to determine whether this cytokine mixture (GM-CSF and IL-3) has protective effects against ischemic rat brain injury caused by transient (90 min) middle cerebral artery occlusion (tMCAO) and also to determine whether the mixture affects the functions of microglia/macrophages in ischemic brains.

## Materials and methods

### Animal maintenance, model preparation, and cytokine injection

All rat experiments were performed in accordance with the Guidelines of the Ethics Committee for Animal Experimentation of Ehime University, Japan (approval number: 05-RU-7-1). Wistar rats (Clea Japan, Tokyo, Japan) were bred and housed (four rats/cage) with a 12-h light/dark cycle (lights on 7:00–19:00) at the animal facility, Ehime University, where food and water were provided *ad libitum*. All invasive procedures were carried out under inhalation anesthesia using 2.0% isoflurane (Mylan Pharmaceutical Company, Tokyo, Japan), and brain tissues were sampled after rats were euthanized by CO_2_ inhalation exposure (Matsuyama Nishi Sanso Company, Matsuyama, Japan).

Adult male Wistar rats (8 weeks old; body weight, 260–280 g) were selected for tMCAO, as described in our earlier reports (Matsumoto et al., 2015; Islam et al., 2018). In brief, under deep anesthesia, the right middle cerebral artery (MCA) was occluded using an intraluminal filament approach, and a 4-0 nylon monofilament suture was inserted from the right external carotid artery into the internal carotid artery. After placing the intraluminal filament, the neck incision was sutured, and the rats in the home cage were replaced for 90 min. The rats were then anesthetized again, and the intraluminal suture was carefully removed. Based on our report on TBI and PD, a cytokine mixture containing 0.2 mg/mL rat recombinant GM-CSF (PeproTech, London, United Kingdom) and 0.2 mg/mL rat recombinant IL-3 (PeproTech) was subcutaneously injected the day after occlusion at a daily dose of 10 μg/kg body weight (Choudhury et al., 2011; Nishihara et al., 2011). Five doses of the cytokine mixture were given for macroscopic and behavioral studies, but only two doses were given for other studies.

### Macroscopic measurements of transient middle cerebral artery occlusion lesion size

Using 1.5 T MRminiSA (DS Pharma Biomedical, Osaka, Japan), magnetic resonance (MR) imaging was performed at days post-reperfusion (dpr) 1, 10, 30, 60, and 90. A T2-weighted MR sequence was used with 1-mm slice thickness (total 12 slices). Following MR imaging, the rat brains were dissected out and stored in phosphate-buffered saline containing 4% paraformaldehyde (PFA, Wako, Osaka, Japan) for 7 days. Then, four 2-mm-thick slices (from anterior, 2nd to 6th) were cut from each brain and photographed. The lesion area of nine MRI images and four PFA fixed slices were quantified as described in our earlier report (Nishioka et al., 2016).

### Behavioral tests

Animals underwent neurobehavioral assessment by a blinded assessor 1 month after surgery. All tests were done at the same time and were counter-balanced.

### Morris water maze test

Spatial learning and memory were evaluated in the Morris water maze. The maze consisted of a video-tracking system (Ethovision XT 7)-equipped, 150-cm-diameter × 45-cm-deep circular pool filled with water at 25°C. A 12-cm-diameter circular transparent platform was placed 2 cm above the waterline at the center of one quadrant. The rats were trained to find this platform over 2 days of training trials (three trials per day) by releasing them in the opposite quadrant of the platform. On the third day, a probe trial of spatial memory was performed in which the platform was submersed 2 cm, and the rats were allowed to swim freely in water for 90 sec. Considering the anxiety behavior of model rats, clear water was used in the current study to avoid the effects of anxiety on cognitive function tests. The swim distance was recorded as an index of spatial memory (Abe et al., 2018).

### Rota-rod test

The rota-rod test (Rota-rod 7750, Ugo Basile, Italy) was implemented in which the speed of the rota-rod was accelerated from 4 to 20 rpm in 1 min. The rats were trained for 3 days before the test. The day after completion of the training, the test was performed, and data were calculated as described previously (Taguchi et al., 2019).

### Open-field test

The open-field test was conducted in a 100 × 100 cm^2^ open and black-floored field surrounded by black 45-cm high walls and equipped with a video-tracking system (Ethovision XT 7). A rat was placed at the center of the corner of the field, and a video of movement was recorded for 5 min. For anxiety measurement, only the duration of time spent in the 60 × 60 cm^2^ center zone was measured (Taguchi et al., 2019).

### Immunofluorescence

Rats were transcardially perfused with 4% PFA. The dissected brains were immersed in 15% sucrose in phosphate-buffered saline at 4°C for 7 days. After sucrose treatment, 10-μm-thick, coronal frozen sections were sliced at the caudoputamen level (Matsumoto et al., 2007). The brain sections were incubated with primary antibodies (mouse Bcl-xL, Transduction Laboratories, Lexington, KY; rabbit Iba1, Wako; and Guineapig NeuN, Mark Millipore, Burlington, MA) and secondary antibodies (DyLight 488-, DyLight 549-, DyLight 649-labeled donkey secondary antibodies, Jackson ImmunoResearch Laboratories, West Grove, PA). The immunostained specimens were visualized with a Nikon A1 confocal laser scanning microscope. The low magnification image is shown in Supplementary Figure 1 for pointing the area of the brain from which the immunofluorescent image was derived. Images of positive control (tumor tissue) and negative controls (healthy tissue), no primary antibody controls for Bcl-xL are shown in Supplementary Figure 2 (Karmakar et al., 2007; Ozaki et al., 2021).

### Fluorescence-activated cell sorting and quantitative polymerase chain reaction

The ipsilateral hemispheres of rat brains were processed and dissociated as described in our paper (Abe et al., 2018). The dissociated cells were stained with antibodies (APC mouse anti-rat CD11b, BD Bioscience, San Jose, CA; PE mouse anti-rat granulocytes, BD Bioscience; and Alexa700 mouse anti-rat CD45, BioLegend, San Diego, CA), and the stained cells were treated and stored at 4°C with cell cover (AL ANACYTE, Hamburg, Germany) until sorting. Before staining, cells were incubated on ice with a mouse anti-rat CD32 antibody (BD Bioscience) for 20 min to block Fc receptors. Granulocyte-negative, CD11b-positive, and CD45-positive cells were considered microglia and macrophages. CD45-high cells and CD45-low cells were sorted using a fluorescence-activated cell sorting (FACS) Aria III (BD Biosciences) with a 100-μm nozzle and BD FACSDIVA software (BD Biosciences) and considered macrophages and microglia, respectively; sorted cells were processed for RNA extraction. Viable cells were identified using Zombie NIR (BioLegend). RNA extraction and cDNA preparation from sorted microglial cells were performed using a SuperPrep II cell Lysis & RT Kit for quantitative polymerase chain reaction (qPCR) (Toyobo, Osaka, Japan) according to the manufacturer’s instructions. qPCR analysis was performed as described in our previous report (Islam et al., 2018) using qPCR primers (Bcl-xL: forward CCTATCTTGGCTTTGGATCC, reverse TTTCTTCTGGGGCTTCAGTC; CD86: forward CTCAGTGATCGCCAACTTCA, reverse CTGCATGTTGTCGCCATACT; CD163: forward AGCGTCTCTGCTGTCACTCA, reverse CGTTCATGCTCCCAGCCGTTA; IL-1β: forward CACCTTCTTTTCCTTCATCTTTG, reverse GTCGTTGCTTGTCTCTCCTTGTA; tumor necrosis factor [TNF]-α: forward AAAGCATGATCCGAGATGTG, reverse AGCAGGAATGAGAAGAGGCT; and GAPDH: forward GAGACAGCCGCATCTTCTTG, reverse TGACTGTGCCGTTGAACTTG; Hokkaido System Science Co., Ltd., Hokkaido, Japan).

### Immunoblotting

Tissue sampling was done at the following regions: (A) contralateral (contra), (B) surrounding area (peri), and (C) core (core) at 3 dpr (Matsumoto et al., 2007). Laemmli’s sample solution was used for sample preparation and immunoblotting using mouse monoclonal antibodies (β-actin, Wako; and Bcl-xL, Transduction Laboratories), as described previously (Islam et al., 2018).

### Flow cytometry

As above, ipsilateral hemispheres were processed for flow cytometry (FCM) using antibodies (APC mouse anti-rat CD11b, BD Bioscience; Alexa700 mouse anti-rat CD45, BioLegend; Pacific blue mouse anti-rat granulocytes, BD Bioscience; FITC mouse anti-rat CD163, BioLegend; and PE mouse anti-rat CD86, BioLegend). The cells were analyzed with a Gallios instrument flow cytometer (Beckman Coulter, Tokyo, Japan) and FlowJo software (version 7.6.5, Treestar, Ashland, OR), as described previously (Abe et al., 2018).

### Statistics

Data are expressed as means ± standard deviation (SD). Group means were compared by the two-tailed unpaired Student’s *t*-test or two-way ANOVA with Sidak’s multiple comparisons test. All analyses were performed using Prism 9 (GraphPad Software, La Jolla, CA). *P* < 0.05 was considered significant for all tests.

## Results

### Cytokine treatment reduces lost tissue volume in transient middle cerebral artery occlusion model rats

**FIGURE 1:**
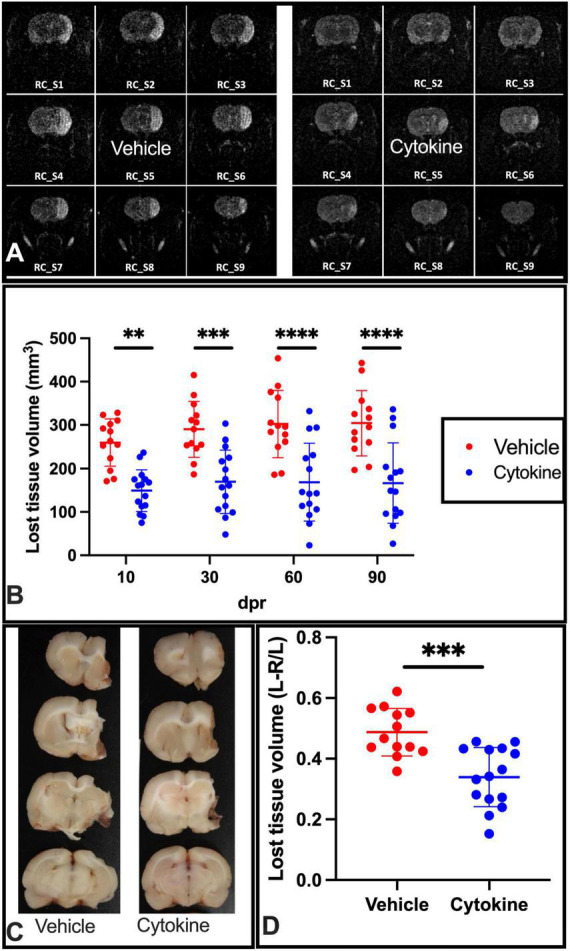
Subcutaneous injection of cytokines (GM-CSF and IL-3) significantly reduces lost tissue volume in ischemic rat brains. **(A)** Representative MR images of vehicle-treated and cytokine-treated rats taken at 90 dpr. **(B)** Time course for MR images. Compared to the vehicle-treated group, the infarct volume (mm^3^) is significantly smaller in the cytokine mixture-treated group. **(C)** Representative coronal brain section images in which the brain was dissected out, fixed in paraformaldehyde, and sliced at 90 dpr. **(D)** Bar graph shows significantly less brain volume loss in the cytokine-treated group than in the vehicle-treated group. Data (*n* = 13–15) are shown as means ± SD and were statistically analyzed with two-way ANOVA for graph B and Student’s *t*-test for graph D. ^**^P < 0.01, ^***^P < 0.001, ^*⁣*⁣**^P < 0.0001 vs. vehicle. RC_S, rostro-caudal slice; GM-CSF, granulocyte macrophage colony-stimulating factor; IL, interleukin; dpr, days post-reperfusion; MR, magnetic resonance; SD, standard deviation.

Considering the ameliorating effects of the cytokine mixture (GM-CSF and IL-3) that were observed in TBI and PD model rats (Choudhury et al., 2011; Nishihara et al., 2011), the ameliorating effects of these cytokines were assessed in tMCAO model rats when rats were subcutaneously administered the mixture for 5 consecutive days starting from the day after tMCAO. To determine whether the mixture had ameliorating effects on ischemic injuries, a time course study with MR imaging was first performed at 10, 30, 60, and 90 dpr. At 10 dpr, the lost tissue volume was significantly lower than in the saline-treated group (*P* < 0.01). A more marked protective effect of the cytokine treatment was noticed at 30 dpr (*P* < 0.001), 60 dpr (*P* < 0.0001), and 90 dpr (*P* < 0.0001) (Figures 1A,B). Following MRI, the rats were sacrificed, and their brains were sliced to measure the lost brain volumes at 90 dpr. The cytokine treatment prevented ischemia-induced brain tissue loss volume at this time point as well (*P* < 0.0001, Figures 1C,D).

### Ameliorating effects of the mixture on transient middle cerebral artery occlusion-induced behavioral dysfunction

**FIGURE 2:**
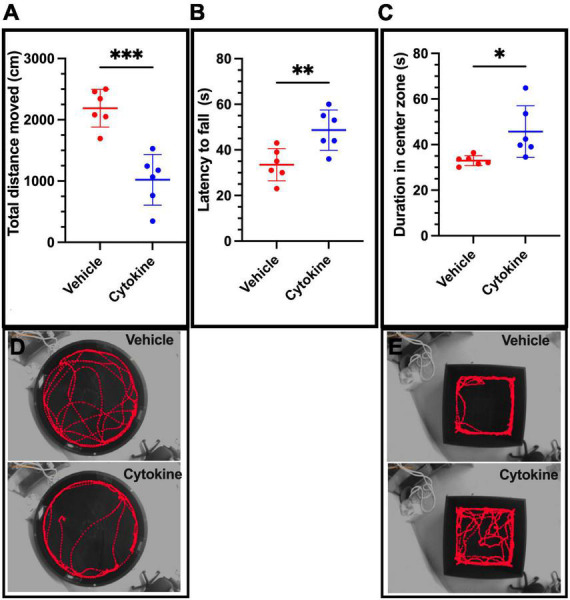
Cytokine (GM-CSF and IL-3) treatment significantly ameliorates neurological functional deficits in ischemic brain rats at 30 dpr. **(A)** Cytokine administration ameliorates spatial cognitive dysfunction, as shown by the Morris Water Maze test. **(B)** Cytokines improve motor deficits as assessed by the rota-rod test. **(C)** On the open-field test, the cytokine-treated group spends more time in the center zone. **(D)** Representative swim traces in Morris Water Maze test. **(E)** Representative travel route maps in the open-field test for the two groups. Data are shown as means ± SD and were statistically analyzed with Student’s *t*-test (*n* = 6). *P < 0.05, ^**^P < 0.01, ^***^P < 0.001 vs. vehicle vs. vehicle. GM-CSF, granulocyte macrophage colony-stimulating factor; IL, interleukin; dpr, days post-reperfusion; SD, standard deviation.

Because tMCAO impairs cognitive function (Li et al., 2022), effects of the cytokine mixture on tMCAO-induced cognitive dysfunction were evaluated by the Morris water maze test. The cytokine mixture ameliorated spatial cognitive function, since the rats administered the cytokine mixture traveled less (*P* < 0.001) to find the platform than saline-treated rats (Figures 2A,D; Abe et al., 2018). tMCAO also impairs motor coordination (Hu et al., 2021), and the cytokine treatment significantly ameliorated tMCAO-induced motor incoordination, because it prolonged latency to fall compared to the saline-treated group in the rota-rod test (*P* < 0.01, Figure 2B; Choudhury et al., 2011). An earlier report showed that the ischemic rats stayed for a shorter period in the open arm in the EPM test than non-ischemic healthy rats, suggesting that the ischemic rats were more anxious than the healthy rats. Furthermore, as shown on the open-field test, the ischemic rats stayed for shorter periods in the center zone, exhibiting anxiety-like behavior (Zhang et al., 2017; Radhakrishnan and Gulia, 2018). The cytokine mixture-treated rats stayed for longer periods in the center zone in the open-field test compared to saline-treated rats (*P* < 0.05, Figures 2C,E). Taken together, these results show that the cytokine mixture improved ischemia-induced cognitive dysfunction and anxiety-like behaviors.

### Increased expression of Bcl-xL in cytokine-treated transient middle cerebral artery occlusion rats

**FIGURE 3:**
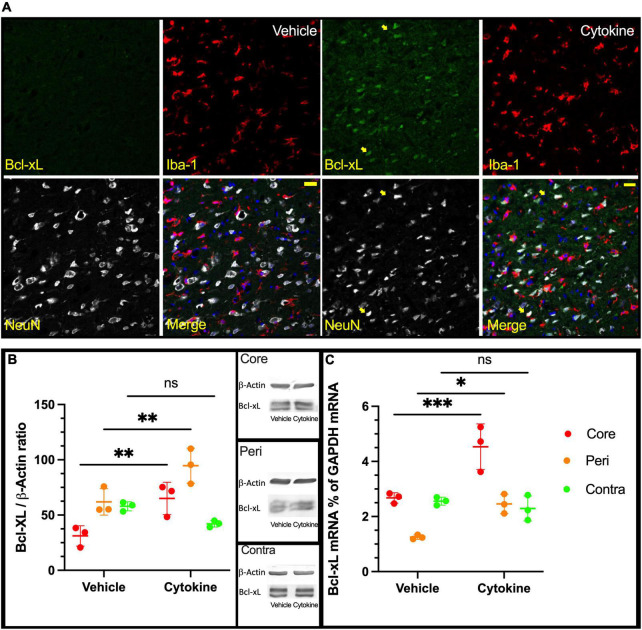
Increased expression of Bcl-xL following cytokine (GM-CSF and IL-3) administration at 3 dpr. **(A)** Representative immunohistochemical data in which Bcl-xL is only faintly expressed in nerve cells of the vehicle-treated group. In the cytokine group, Bcl-xL is prominently expressed in many neurons as indicated by the yellow arrows, scale bar-50 μm. **(B)** Expression of Bcl-xL protein in the core, peri, and contra regions at 3 dpr. **(C)** Expression of Bcl-xL-encoding mRNA at 3 dpr in core, peripheral, and contra tissues. Immunoblotting and qPCR data analyzed with ANOVA and are shown as means ± SD (*n* = 3). *P < 0.05, ^**^P < 0.01, ^***^P < 0.001 vs. vehicle of each group. GM-CSF, granulocyte macrophage colony-stimulating factor; IL, interleukin; dpr, days post-reperfusion; ANOVA, analysis of variance; SD, standard deviation.

Since IL-3 and GM-CSF have been shown to have stimulating effects on the expression of the anti-apoptotic factor Bcl-xL (Choudhury et al., 2011), an immunofluorescence study with antibodies against NeuN, Iba1, and Bcl-xL was done using brain sections from ischemic rats with and without the cytokine mixture treatment at 3 dpr. Bcl-xL was only faintly expressed in neuronal cells in the saline group in the peripheral region, whereas in the cytokine mixture-treated brains, neuronal cells expressed Bcl-xL more abundantly in the same region (Figure 3A). Using tissue samples from the core, peri, and contra regions of 3 dpr, Bcl-xL expression was examined by immunoblotting because the number of live cells did not show a significant difference between the groups at this time (Supplementary Figure 3). Though the cytokine mixture treatment did not induce any significant changes in the contra regions (*P* = 0.2775, Figure 3B), it significantly increased expression of Bcl-xL protein in the peri (*P* < 0.01) and core (*P* < 0.01) regions compared to saline treatment (Figure 3B). Similar notion was seen in mRNA level, where Bcl-xL was most abundantly expressed in cytokine-treated peri (*P* < 0.05, Figure 3C) and core (*P* < 0.001, Figure 3C) tissues compared to saline treatment, but these changes were absent in contra tissue (*P* = 0.8424, Figure 3B).

### Cytokine treatment suppresses microglial expression of pro-inflammatory cytokines in the ischemic rat brain

**FIGURE 4:**
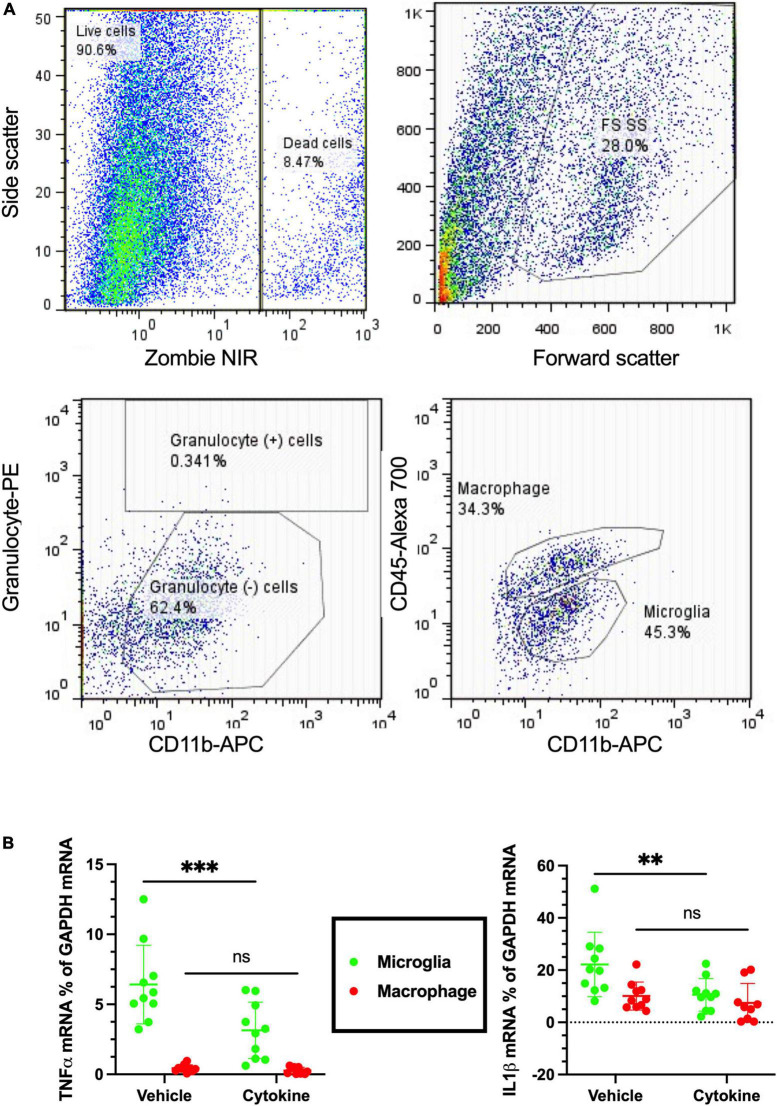
Cytokines (GM-CSF and IL-3) inhibit microglial release of inflammatory cytokines following ischemic injury at 3 dpr. **(A)** Flow cytometry gating strategies in which the ischemic hemisphere at 3 dpr is dissociated and gated for granulocyte, macrophage, and microglia sorting. **(B)** Expression of mRNA by microglia isolated from the ischemic hemisphere by flow cytometry is analyzed by qPCR (*n* = 10). Cytokine treatment significantly inhibits microglial release of TNF-α, and IL-1β, but does not show any changes in macrophages. Data are shown as means ± SD and were statistically analyzed with Student’s *t*-test. ^**^P < 0.01, ^***^P < 0.001 vs. vehicle. GM-CSF, granulocyte macrophage colony-stimulating factor; IL, interleukin; dpr, days post-reperfusion; TNF, tumor necrosis factor; SD, standard deviation.

Neuroinflammation is a common feature following focal cerebral ischemia, and studies have confirmed that neuroinflammatory processes can induce neuronal apoptosis (Hoehn et al., 2005; Liu et al., 2007). Microglia and macrophages are critical cells causing immune responses in the ischemic brain lesions by releasing various pro-inflammatory and anti-inflammatory mediators (Lambertsen et al., 2005; Clausen et al., 2008, 2016). Of the pro-inflammatory mediators, TNF-α and IL-1β are considered to be the most hostile components in stroke brains, as well as blood-derived macrophages and resident microglia whose sources have been reported (Abe et al., 2018). Notably, the 3-dpr ischemic events were characterized by decreased homeostatic microglia, monocyte infiltration, and macrophage transformation, which are considered to be critical for exacerbation of neurodegeneration (Benakis et al., 2014). To see the immunity-related, cellular-specific effects of the cytokines (IL-3 and GM-CSF) on ischemic brain, tissue at 3-dpr was dissociated, blood-borne macrophages (CD45*^hi^*/CD11b^+^ cells) and resident microglia (CD45*^lo^*/CD11b^+^ cells) were sorted with FACS, and mRNA expression was quantified by qPCR (Figure 4A). Cytokine treatment provided significant silencing effects on the mRNA levels of TNF-α (*P* < 0.001), and IL-1β-α (*P* < 0.01) in sorted microglia from the ischemic hemisphere. Nonetheless, contrary to our prediction, their mRNA expression levels on sorted macrophages remained unchanged (Figure 4B). In summary, the present outcomes further indicate that cytokine treatment mediates anti-inflammatory effects by modulating only microglia, but not invaded macrophages.

### Cytokine treatment modulates microglial polarization in the ischemic rat brain

**FIGURE 5:**
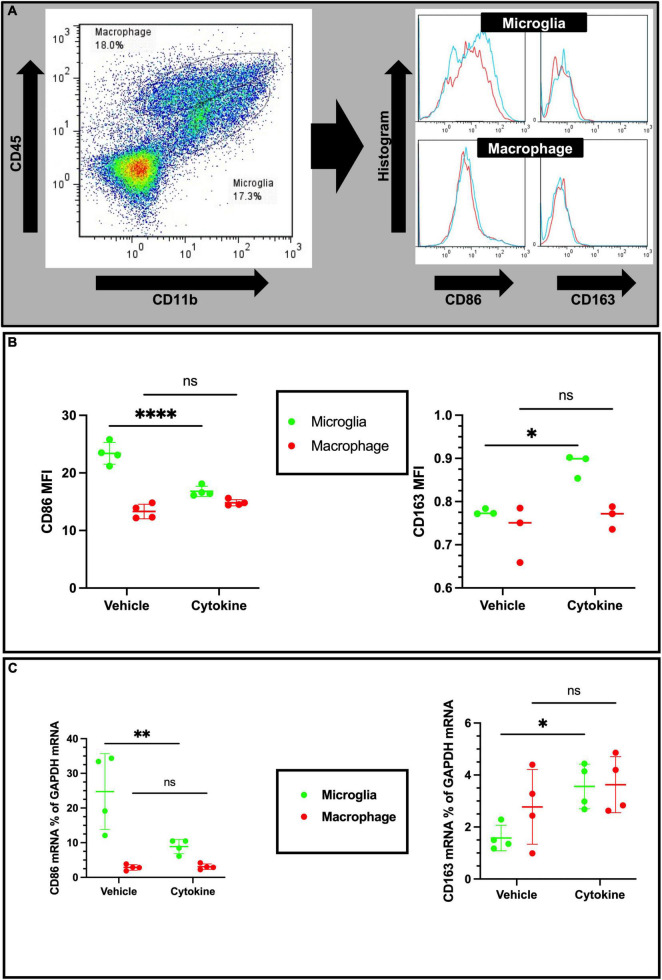
Cytokines (GM-CSF and IL-3) modulate phenotypic changes in the ischemic brain at 3 dpr. **(A)** Gating strategy for flow cytometric analysis. The histogram shows CD86-positive and CD163-positive microglia (above) and macrophages (below). **(B)** Based on mean fluorescence intensity measurement, cytokine treatment decreases expression of CD86 and increases expression of CD163 on microglial cells at protein levels. **(C)** Expression of CD86 and CD163 encoding mRNA in sorted microglia of the ipsilateral hemisphere. Flow cytometry and qPCR data analyzed with ANOVA and are shown as means ± SD (Flow cytometry, *n* = 3; qPCR, *n* = 4). *P < 0.05, ^**^P < 0.01, ^*⁣*⁣**^P < 0.0001 vs. vehicle of each group. GM-CSF, granulocyte macrophage colony-stimulating factor; IL, interleukin; dpr, days post-reperfusion; ANOVA, analysis of variance; SD, standard deviation.

Considering cellular-specific changes of mRNA expressions of pro-inflammatory cytokines, cytokine treatment may have potential modulating effects on the polarization of microglia and macrophages in the ipsilateral part of ischemic brain. In addition, macrophages and microglia also polarize toward pro-inflammatory and tissue-inflammatory types in ischemic brain (Zhang et al., 2021). In addition to CD11b and CD45 antibodies, the antibody marker CD86 for the pro-inflammatory phenotype and CD163 for the tissue-repairing phenotype were used, as described elsewhere (Figure 5A; Li et al., 2018). As anticipated from the qPCR data, the cytokine treatment markedly decreased mean florescence intensity (MFI) for CD86 of microglial cells (*P* < 0.0001), but did not show any effect on infiltrated macrophages (Figure 5B, Left). In accordance with the decreased microglial pro-inflammatory polarization, the expression of the tissue-repairing phenotype marker CD163 on microglial cells was found to increase (*P* < 0.05), but there were no changes on macrophages (Figure 5B, right). In agreement with flow cytometric data, cytokines induced CD86 mRNA suppression (*P* < 0.01) and increased CD163 expression (*P* < 0.05) only in sorted microglia but not in sorted macrophages from the ipsilateral hemisphere of 3 dpr (Figure 5C). Collectively, these findings suggest that cytokine treatment specifically inhibits polarization of microglia toward the pro-inflammatory phenotype and, concomitantly, facilitates polarization of microglia toward the tissue-repairing phenotype.

## Discussion

This study showed that subcutaneous administration of the cytokine mixture containing GM-CSF and IL-3 markedly ameliorated tMCAO-induced brain tissue loss and impairments of cognitive and motor functions. The prolonged ameliorating effects of the cytokine mixture were observed 3 months after tMCAO. The effects may be attributable to the cytokine-induced elevated expression of the anti-apoptotic factor Bcl-xL in neurons in the peripheral region, which may be correlated with prevention of secondary neurodegeneration after the primary ischemic degeneration. The suppressed apoptotic degeneration may be due to cessation of microglial pro-inflammatory polarization.

Both cytokines used in the present study have been separately investigated for their neuroprotective effects using animal models of various neurological disorders. GM-CSF is an astrocyte-derived cytokine in brain that promotes ramification of microglial cells (Giulian et al., 1995; Fujita et al., 1996) and also increases phagocytic activity by microglia (Choudhury et al., 2021). In Alzheimer’s disease brains, IL-3 ameliorates Alzheimer’s disease pathology and cognitive dysfunction, while promoting microglial motility (McAlpine et al., 2021). In our previous study with a 6-hydroxydopamine-induced rat PD model, it was found that microglia express higher levels of the GM-CSF receptor than neurons, and neurons express higher levels of the IL-3 receptor than microglia. More interestingly, when primary cultured microglia were treated with both GM-CSF and IL-3, they increased release of neurotrophic factors, as well as phagocytic ability (Choudhury et al., 2011, 2021).

Intracarotid injection of GM-CSF increases the number of activated microglia/macrophages and also inhibits cellular apoptosis in the ischemic penumbra after MCAO (Nakagawa et al., 2006). GM-CSF in MCAO model rats shows a direct protective effect on neurons by increasing the expression of anti-apoptotic proteins (Schabitz et al., 2008), and subcutaneous treatment with GM-CSF also ameliorates MCAO-induced motor dysfunction (Dames et al., 2018). In addition to GM-CSF, astrocytes constitutively produce IL-3 in brain, which provokes morphological changes and phagocytic activity of microglia (Suzumura et al., 1990; Fujita et al., 1996; Choudhury et al., 2011; Nishihara et al., 2011). IL-3 is neuroprotective in the ischemic brain and increases expression of Bcl-xL and ameliorates MCAO-induced cognitive dysfunction (Wen et al., 1998). Although, the number of viable cells in the cytokine-treated and untreated groups at our current study are similar at 3 dpr of tMCAO rats, this treatment showed significant improvement on tissue loss as we started to notice from 10 dpr. As early as 3 dpr, cytokines increased Bcl-xL expression at the site of injury which can contribute to the process of improving tissue loss and behavioral dysfunction.

In the ischemic brain, microglia have been regarded as double-edged swords because they mediate cytotoxic and/or cytoprotective effects by releasing pro-inflammatory and anti-inflammatory factors (Patel et al., 2013). In addition, mounting evidence suggests that microglial phagocytosis is a mixed blessing to neuroinflammation and tissue regeneration. Microglia phagocytose live cells that cause neuronal cell death and blood-brain barrier leakage. On the other hand, microglial phagocytosis involves inflammatory processes through engulfing cell debris, scrubbing infiltrating neutrophils, and forming the best microenvironment for neurogenesis (Jia et al., 2021). Even though microglia are neuroprotective for ischemic brain, microglia are very vulnerable to ischemic conditions, and they degenerate shortly after ischemic insults (Matsumoto et al., 2007). However, depletion of microglia by using the chemical PLX3397, a colony-stimulating factor 1 receptor inhibitor, exacerbates ischemic injury while augmenting leukocyte infiltration and the astrocyte response (Jin et al., 2017). Therefore, microglia are neuroprotective cells rather than neurodestructive ones in ischemic brain. However, in accordance with our previous report describing the neuroprotective effects of reactive microglia in traumatic brain lesions, neighboring invaded macrophages are able to respond to ischemic insults by increasing production and release of pro-inflammatory mediators, which are potentially harmful to viable peripheral regions (Abe et al., 2018). Therefore, such interventions are necessary to augment the neuroprotective actions of microglia and at the same time to suppress pro-inflammatory reactions for brain ischemic patients to prevent ischemia-induced secondary neurodegeneration. In this study, changes in microglial expression between CD86, an inflammatory cell marker, and CD163, a tissue remodeling cell maker, differ between the cytokine-treated and untreated groups and the shift in cell phenotype makers likely contributed to reduce ischemic injury and improved behavioral outcomes in the treated group (Li et al., 2018).

**FIGURE 6:**
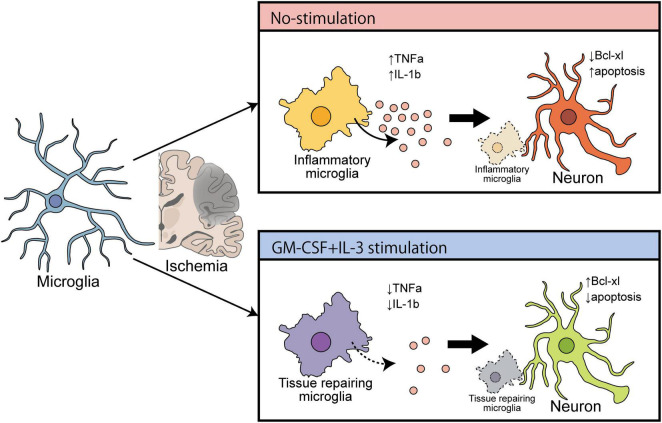
Schematic diagram showing the ameliorating effect of cytokines (GM-CSF and IL-3) on cerebral ischemia that induces brain recovery by converting inflammatory microglia into a tissue repair type.

Accumulating evidence suggests that remodeling microglia to a protective phenotype is hypothetically effective in treating neurodegenerative diseases such as PD (Awogbindin et al., 2020). However, following ischemic brain injury, the expressions of IL-1β and TNF-α were drastically increased in the microglia in the ipsilateral region, as shown by combined FACS and qPCR (Wang et al., 2020). Microglia-derived TNF-α induces apoptosis in neuronal progenitor cells by increasing expression of pro-apoptotic genes that belong to the Bcl-2 family protein Bax *via* an NF-κB-dependent mechanism (Guadagno et al., 2013). Furthermore, microglia-derived IL-1β induces cellular apoptosis by targeting p53 (Guadagno et al., 2015). Therefore, neuronal apoptosis in the ischemic brain may be due to endogenous release of cytokines from either microglia or adjoining macrophages. In the present study using tMCAO model rats at 3 dpr, the brain immune cell-specific effects of cytokine mixtures that specifically decreased expressions of TNF-α, and IL-1β on microglia, but not macrophages, were examined. Similar ameliorating effects of cytokine mixtures containing GM-CSF and IL-3 have been demonstrated against TBI and PD (Choudhury et al., 2011; Nishihara et al., 2011). Thus, it is plausible that cytokine treatment has an anti-apoptotic action in ischemic rat brain by inhibiting microglial expression of pro-inflammatory mediators. Furthermore, the bolstering effects of cytokine treatment on microglial morphology and phagocytosis have been documented in the previous report from our lab (Fujita et al., 1996; Choudhury et al., 2021), and this study is the first to present the polarizing effect of cytokine treatment on microglia. Using a hypothesis from a recent review about microglial polarization in the ischemic brain (Zhang et al., 2021) and the evidence from the present study, it appears that microglial remodeling can be effective in treating the ischemic brain (Figure 6).

## Conclusion

In the ischemic brain, treatment with a cytokine mixture containing GM-CSF and IL-3 may be worth trying to prevent ischemia-induced secondary neurodegeneration. The cytokine mixture prevents neuronal apoptosis by increasing Bcl-xL expression by still viable neurons and reactive microglia in the peripheral region. Suppression of pro-inflammatory activation of microglia probably further leads to suppression of neuronal apoptosis and ameliorates ischemic brain injury. However, how cytokine cause microglial polarization toward tissue repairing type from pro-inflammatory type necessitate future studies to specifically look at different signaling pathways involved with the polarization process.

## Data availability statement

The raw data supporting the conclusions of this article will be made available by the authors, without undue reservation.

## Ethics statement

The animal study was reviewed and approved by the Ethics Committee for Animal Experimentation of Ehime University. Written informed consent was obtained from the owners for the participation of their animals in this study.

## Author contributions

JT developed the concept and led the study. SM prepared animal models and analyzed MR imaging data and behavioral studies. MC performed drug administration, MR imaging, behavioral experiments, FCM, FACS, and immunohistochemistry. HT performed qPCR with sorted samples. NK, AS, and KM performed immunoblotting. AI, HY, HW, and TK provided intellectual input for the study. SM and MC wrote the manuscript. YK and JT revised and finalized the manuscript. All authors read and approved the final version of the manuscript.

## Abbreviations

GM-CSF: granulocyte macrophage colony-stimulating factor
IL: interleukin
dpr: days post-reperfusion
MR: magnetic resonance
TNF: tumor necrosis factor
SD: standard deviation
ANOVA: analysis of variance.
