# Supplementary material for: Microglial re-modeling contributes to recovery from ischemic injury of rat brain: A study using a cytokine mixture containing granulocyte-macrophage colony-stimulating factor and interleukin-3
Source: Front Neurosci. 2022 Jul 28;16:941363. doi: 10.3389/fnins.2022.941363 (PMC9366522; doi:10.3389/fnins.2022.941363)
Supplement: Supplementary file 1 [file Presentation_1.pdf]

Microglial re-modeling contributes to recovery from ischemic injury of rat brain: A study using a cytokine mixture containing granulocyte-macrophage colony-stimulating factor and interleukin-3

SUPPLEMENTARY MATERIAL

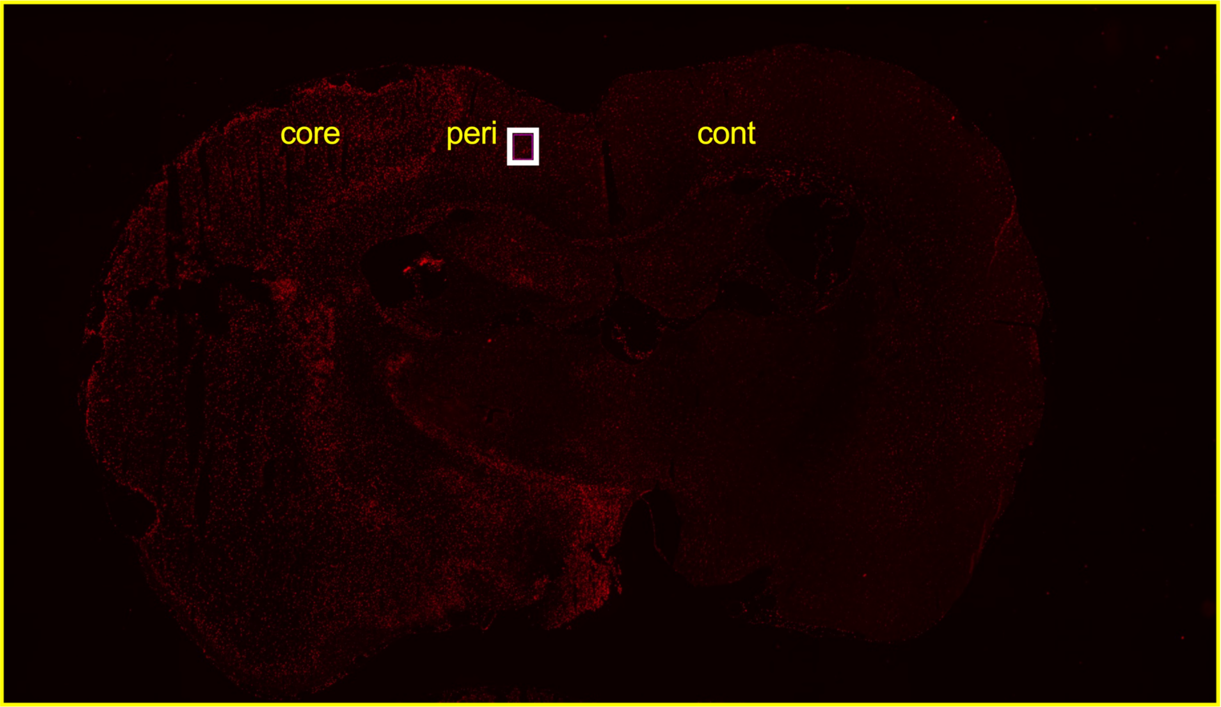

Supplementary Figure 1

Low magnified coronal section of transient middle cerebral artery occlusion (tMCAO) model rat brain at 3 days post re-perfusion (3dpr) where framed area was selected for immunofluorescence images study in Figure 3 of main text.

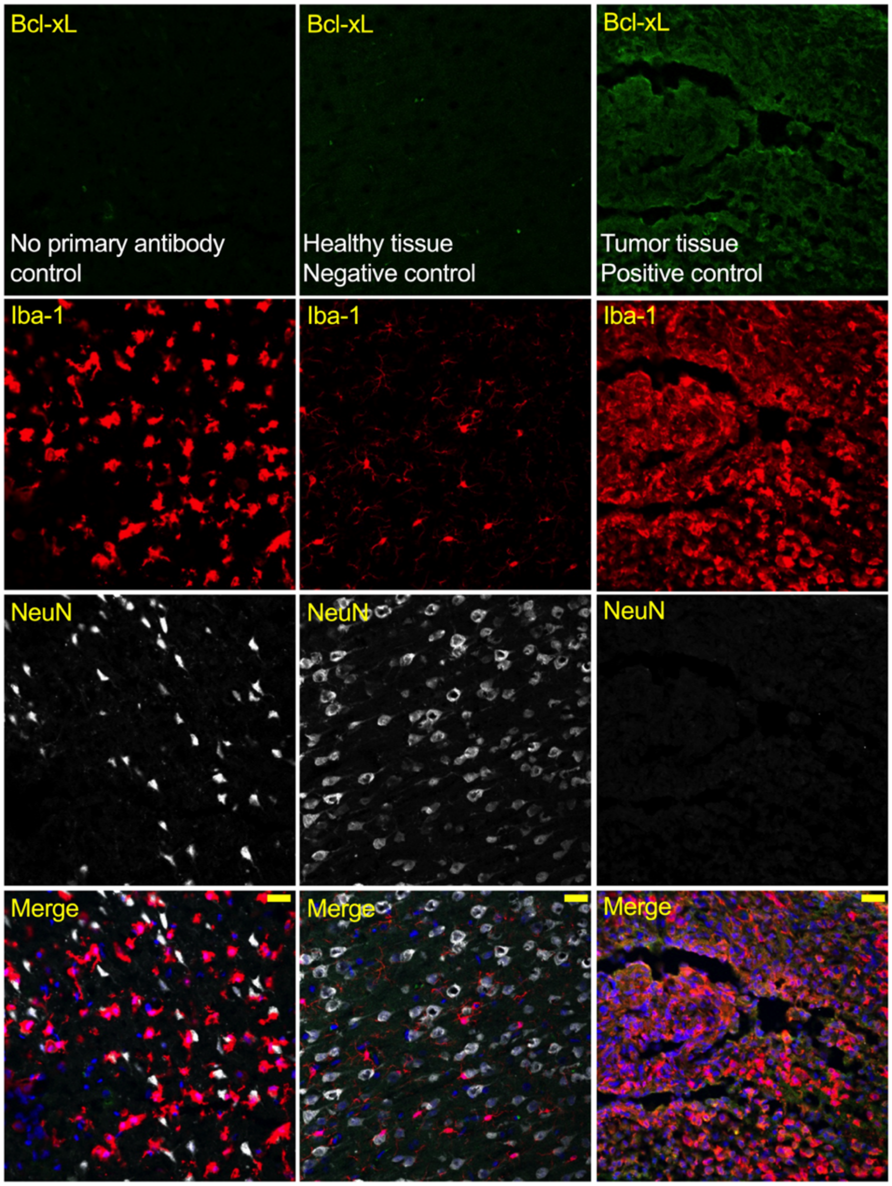

Supplementary Figure 2

Images showing no antibody control, negative, and positive for Bcl-xL immunostaining: Images from tMCAO-induced brain at the region indicated by framed area of supplementary Figure 1 (left) as no antibody control, images at the region of somatosensory cortex from healthy rat brain (middle) as negative control, and images at same region from rat glioma model rat brain tissue (right) as positive control, and. The image settings were the same as those used to collect the images in Fig. 3-A of this manuscript, scale bar-50mm.

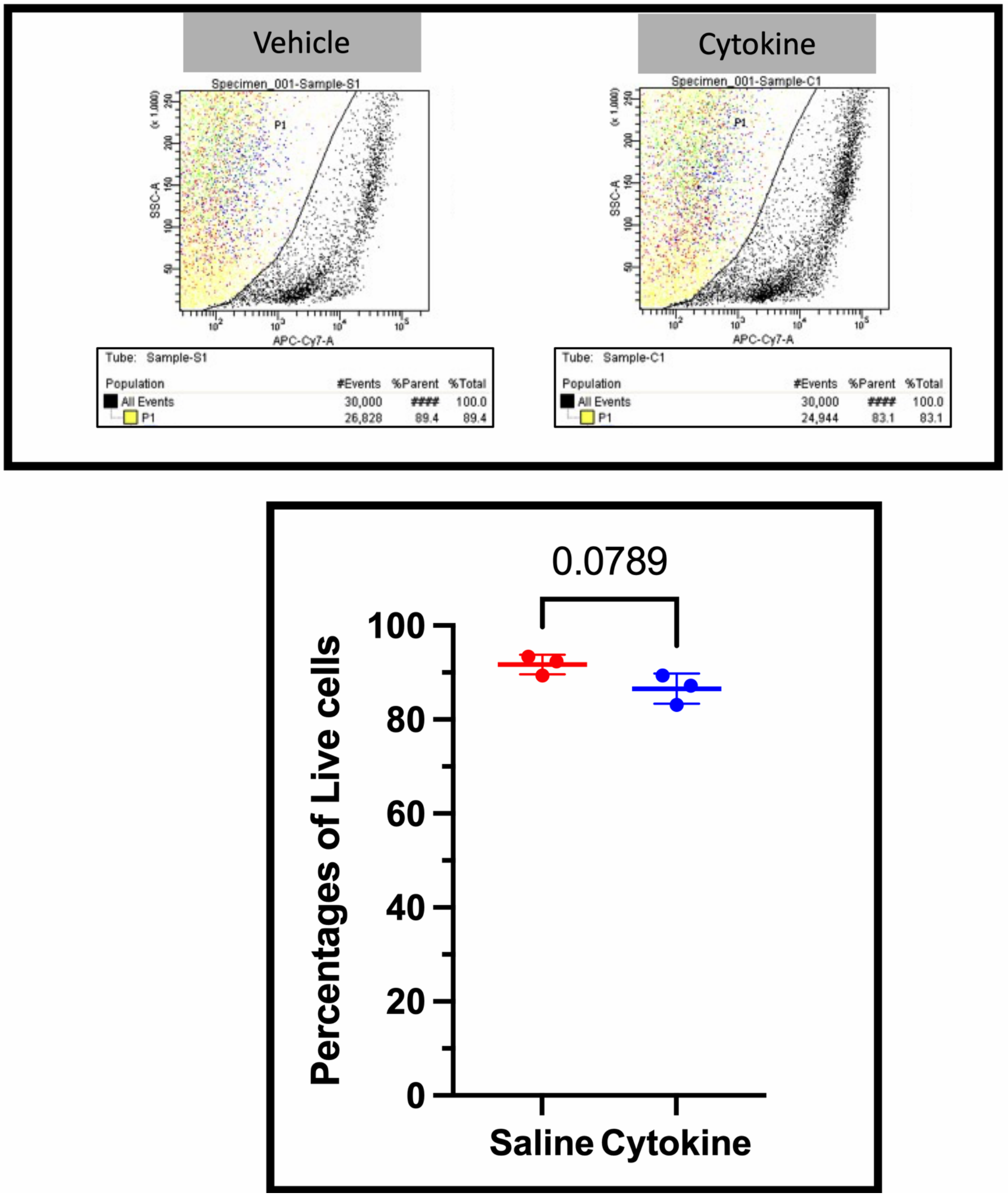

Supplementary Figure 3

Flow cytometric analysis for assessing number of live cells at 3 days post-reperfusion (3dpr) where APC-Cy7 were used for Zombie NIR to discriminate live and dead cells.
